# The Predictive Role of the Biomarker Kidney Molecule-1 (KIM-1) in Acute Kidney Injury (AKI) Cisplatin-Induced Nephrotoxicity

**DOI:** 10.3390/ijms20205238

**Published:** 2019-10-22

**Authors:** Daniela Maria Tanase, Evelina Maria Gosav, Smaranda Radu, Claudia Florida Costea, Manuela Ciocoiu, Alexandru Carauleanu, Cristina Mihaela Lacatusu, Minela Aida Maranduca, Mariana Floria, Ciprian Rezus

**Affiliations:** 1 Department of Internal Medicine, “Grigore T. Popa” University of Medicine and Pharmacy, 700111 Iasi, Romania; tanasedm@gmail.com (D.M.T.); ciprianrezus@yahoo.com (C.R.); 2 Internal Medicine Clinic, “Sf. Spiridon” County Clinical Emergency Hospital Iasi, 700115 Iasi, Romania; sminelaaida@yahoo.com; 3 Department of Cardiology, “Grigore T. Popa” University of Medicine and Pharmacy, 700111 Iasi, Romania; radu.smaranda@gmail.com; 4 Cardiology Clinic, “Prof. Dr. George I.M. Georgescu” Institute of Cardiovascular Diseases, 700503 Iasi, Romania; 5 Department of Ophthalmology, “Grigore T. Popa” University of Medicine and Pharmacy, 700115 Iasi, Romania; costea10@yahoo.com; 6 2nd Ophthalmology Clinic, “Prof. Dr. Nicolae Oblu” Emergency Clinical Hospital, 700115 Iași, Romania; 7 Department of Pathophysiology, Faculty of Medicine, “Grigore T. Popa” University of Medicine and Pharmacy, 700115 Iasi, Romania; mciocoiu2003@yahoo.com; 8 Department of Obstetrics and Gynecology, “Grigore T. Popa” University of Medicine and Pharmacy, 700111 Iasi, Romania; drcarauleanu@yahoo.com; 9 Unit of Diabetes, Nutrition and Metabolic Diseases, “Grigore T. Popa” University of Medicine and Pharmacy, 700115 Iasi, Romania; cmlacatusu@yahoo.co.uk; 10 Clinical Center of Diabetes, Nutrition and Metabolic Diseases, “Sf. Spiridon” County Clinical Emergency Hospital, 700111 Iasi, Romania; 11 Department of Physiology, “Grigore T. Popa” University of Medicine and Pharmacy, 700111 Iasi, Romania

**Keywords:** kidney injury molecule-1, acute kidney injury, cisplatin nephrotoxicity

## Abstract

Acute kidney injury (AKI) following platinum-based chemotherapeutics is a frequently reported serious side-effect. However, there are no approved biomarkers that can properly identify proximal tubular injury while routine assessments such as serum creatinine lack sensitivity. Kidney-injury-molecule 1 (KIM-1) is showing promise in identifying cisplatin-induced renal injury both in vitro and in vivo studies. In this review, we focus on describing the mechanisms of renal tubular cells cisplatin-induced apoptosis, the associated inflammatory response and oxidative stress and the role of KIM-1 as a possible biomarker used to predict cisplatin associated AKI.

## 1. Introduction

The growing incidence of kidney disease is a concern for the scientific community [1]. Recent figures estimate that one in ten Europeans suffer from chronic kidney disease (CKD). The fact that patients in advanced stages of CKD are frequently asymptomatic requires an efficient screening to ensure early detection, especially in high-risk individuals. Moreover, acute kidney injury (AKI) in itself is a risk factor for CKD and has been reported in 20% of hospitalized Europeans. This interplay between AKI and CKD, as well as their association with multiple other comorbidities urges the development of efficient prevention strategies, the importance of an early diagnosis, and appropriate management [2]. 

In this setting, drug-induced nephrotoxicity is a frequently-encountered entity, especially in diabetic patients who associate cardiovascular diseases and a pre-existing degree of renal dysfunction and/or a septic process. Toxic compounds of various pharmacological agents may accumulate and determine mild to moderate nephrotoxicity and subsequent homeostasis disbalance [3,4,5].

Potentially nephrotoxic agents including various drugs, contrast agents or herbal/natural products may directly or indirectly lead to complications [6]. The use of least one nephrotoxic agent has been reported in 25% of AKI cases [7,8,9]. Some of these complications include interstitial nephritis, nephrotic syndrome, and tubulointerstitial disease. The subsequent changes in intraglomerular hemodynamics and the accompanying renal tubular inflammation lead to acute/chronic renal injury and the development of acid-base and/or fluid-electrolyte disturbances. It follows that both nephrotoxic drugs/drugs association and early identification of these effects once developed must be known in order to prevent end-stage renal disease [10]. However, the nephrotoxic process is complex and involves a combination of factors, as not all patients develop kidney disease despite various degrees of exposure [5]. Drug concentration and pharmacokinetics, therapy intensity, and patient’s genetics regarding metabolism and transport influence the probability of developing drug-induced nephrotoxicity. Patient’s age (>65 years), gender (female), associated conditions including metabolic complications and urine pH, as well as the genetics determining the immune response intensity are other reported risk factors [11]. 

Clinically, AKI is defined by oliguria accompanied by a rise in serum creatinine (sCr) within 48 h of either ≥26.5 μmol/L (0.3 mg/dL) or ≥50% as compared to baseline values during the first week of exposure to a potentially nephrotoxic agent [12,13]. Taking into consideration that most drugs are renally cleared, choosing between different agents and adjusting therapy regimens according to the glomerular filtration rate (GFR) is necessary. Antibiotics such as β-lactams, aminoglycosides, vancomycin, amphotericin and sulfonamides, anti-virals (acyclovir) non-steroidal anti-inflammatory drugs, antineoplastic and immunomodulatory (methotrexate, cyclosporin, cisplatin, tacrolimus), and antihypertensives (angiotensin-converting-enzyme inhibitors and angiotensin receptor blockers) are among the most frequently encountered nephrotoxic drugs. Importantly, the nephrotoxic effect may limit the prescription of some agents, especially antineoplastic and various chemotherapeutics [14]. Unfortunately, routine assessments such as blood urea nitrogen (BUN), sCr, GFR, and creatinine clearance become altered late during the course of the disease. Thus, new biomarkers ensuring early detection of kidney disease/injury are needed [15].

As neoplasia is a global issue, the numerous side-effects (neurotoxicity, ototoxicity, and nephrotoxicity) limiting the use of chemotherapy raises concerns. 

One of the most used chemotherapy drugs are platinum-derivatives such as cisplatin [16] oxaliplatin [17,18,19], and carboplatin [20]. Lately, researchers focused on identifying a selective biomarker that can be used to identify and monitor early kidney injury, since current widely used assays lack sensitivity and specificity. Early identification of nephrotoxicity will improve current point-of-care by enabling dose and regimens adjustments before the development of overt renal dysfunction. Potential biomarkers include neutrophil gelatinase-associated lipocalin (NGAL), kidney injury molecule-1 (KIM-1), tissue inhibitor of metalloproteinases-2 (TIMP-2) and insulin-like growth factor-binding protein 7 (IGFBP7). Among these, kidney injury molecule-1, formerly known as T cell immunoglobulin mucin-1 (TIM-1) or hepatitis A virus cellular receptor 1 (Havcr1) [21] and mRNAs have proven to be sensitive for early renal injury both in humans and animal subjects [22,23,24].

In this review, we discuss the mechanisms behind cisplatin-induced nephrotoxicity and the implications of kidney injury molecule-1 (KIM-1) as a biomarker in its early detection, as well as future research perspectives related to the topic.

## 2. Kidney Injury Molecule-1 and AKI

### 2.1. What Is KIM-1?

After extensive research in the field, The Food and Drug Administration FDA and European Medicines Agency (EMEA) approved seven new biomarkers used for nephrotoxicity detection that may influence clinical decision making: KIM-1, albumin, B2-microglobulin, cystatin C, total protein clusterin, and trefoil factor-3 [25,26,27]. Of these, urinary KIM-1 has been approved for drug induced proximal tubular injury identification and monitoring in both animal and clinical studies [3]. KIM-1 is a proximal tubule apical transmembrane protein. Its extracellular component includes O-glycosylated mucin and 6-cysteine domains (the latter with a structure resembling immunoglobulins) [28]. KIM-1 was initially identified using a polymerase chain reaction (PCR). In humans, this molecular family (KIM/TIM) includes three such glycoproteins, as opposed to eight in rodents [29,30] and its levels are very low under normal conditions. However, 48 h after ischemia–reperfusion injury occurs, KIM-1 may be identified in the undifferentiated proliferating proximal tubule epithelial cells [31]. Elevated KIM-1 levels have correlated with inflammation and fibrosis in histological studies [32]. 

In ischemic/toxic renal injury, its extracellular domain is separated from the membrane in a process dependent of a matrix-metalloproteinase (MMP), thus accounting for the increased urinary levels. In AKI patients, the ectodomain shedding leads to a 100-fold increase in urinary KIM-1 levels [23]. However, further research is needed to explore the full pathophysiological implications of KIM-1 extracellular domain shedding [33,34]. 

**Figure 1:**
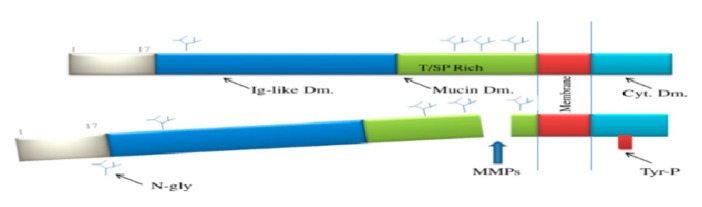
Kidney injury molecule-1 structure. Immunoglobulin-like domain (Ig-like Dm), N-glycosylation sites (N-gly), mucin domain (Mucin Dm), metalloproteinase (MMPs), cytoplasmic domain (Cyt Dm), and P–tyrosine (Tyr–P) site.

Interestingly, KIM-1 has a phosphatidylserine receptor which enhances apoptotic bodies and necrotic debris phagocytosis. This molecule is, therefore, unique for giving epithelial cells a function characteristic of phagocytes [35,36] (Figure 1). It seems that KIM-1 downregulates proximal tubular cell PTC cytokine secretion, modulates translational changes through nuclear factor kappa-light-chain-enhancer of activated B cells (NF-κB) pathway and interaction with phosphatidylinositol3 PI3 kinase subunit p85 [37,38]. Experimentally, KIM-1 gene expression reflects ongoing damage in various tubulointerstitial segments and in the renal cortex [22]. For these reasons, authors began considering KIM-1 a biomarker capable of identifying early AKI and may even hold a possible predictive role [39].

### 2.2. Kidney Molecule-1 in AKI

**Figure 2:**
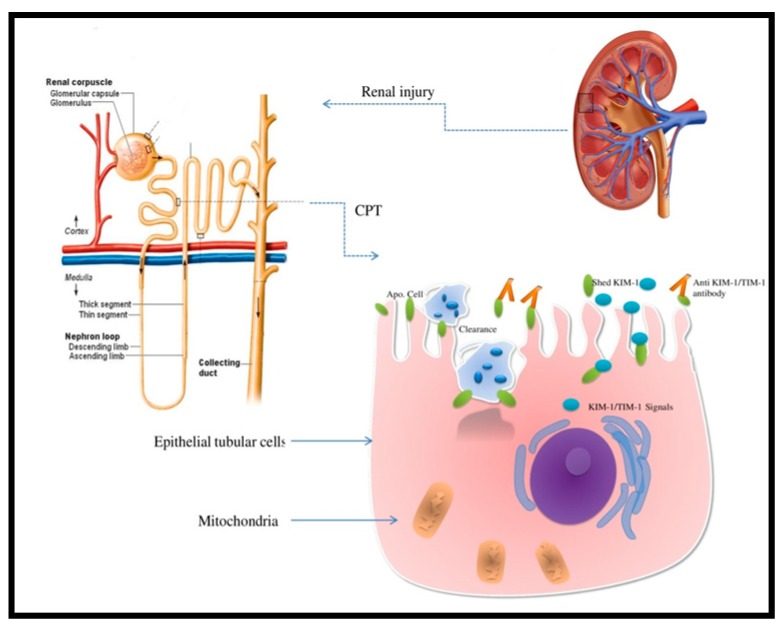
Kidney injury molecule-1 (KIM-1)/T cell immunoglobulin mucin-1 (TIM-1) expression in the proximal convoluted tubule (CPT) after renal injury phagocyting apoptotic cells. The extracellular domain is shed and the phosphatidylserine receptor enhances apoptotic bodies and necrotic debris phagocytosis.

In injured renal cells, KIM-1 may function as a scavenger and phosphatidylserine type-1 receptor overseeing apoptotic cells phagocytosis [40] (Figure 2).

Following a renal injury (either ischemic or toxic), elevated KIM-1 levels may help differentiate acute tubular necrosis (ATN) from prerenal azotemia and CKD. Different authors proposed that elevated KIM-1 levels may also be used to identify patients at risk for progressing from AKI to CKD, based on the observation that levels are constantly elevated in the latter [34,41,42,43]. 

Moreover, some authors state that elevated KIM-1 levels may precede histological changes in AKI patients [44]. However, it must be emphasized that these levels vary with different etiologies [45]. A meta-analysis including 11 studies and 2979 patients estimated urinary KIM-1 specificity in diagnosis of AKI at 86.0% and sensitivity at 74.0% [46]. 

In a different study including 4750 patients followed for more than 10 years, elevated KIM-1 levels correlated with a decline in eGFR, suggesting that this biomarker may be used to predict renal function deterioration in healthy middle-aged patients [47]. In predicting AKI, authors reported an area under the receiver operator curve (AUC) of 0.93 for KIM-1 and interleukinIL-18 [48]. Interestingly, male rats with AKI showed greater increase in KIM-1 levels as compared to females [49,50].

Recent studies explored the ability of KIM-1 to diagnose early and/or predict AKI development in special populations. Urinary KIM-1 in addition to N-acetyl-b-D-glucosaminidase (NAG) and NGAL showed promise in predicting renal injury post-cardiac surgery [48,51]. In decompensated liver cirrhosis patients, AKI may be early diagnosed by combining several biomarkers, such as KIM-1, NGAL, and serum cysteine C (Cys C) [52]. Elevated levels have also been identified in diabetic nephropathy [53], while in transplanted patients it may help in early detection of allograft rejection associated AKI [54,55]. Notably, KIM-1 is overexpressed in renal cell carcinoma, therefore its extracellular domain can be detected in the urine of these patients [56]. 

Taking into consideration its specificity and sensitivity in early detection of renal injury, this biomarker may be used to detect renal dysfunction caused by nephrotoxic drugs [26]. Since various etiologies can account for AKI and that its incidence is increasing, further studies are required to validate new biomarkers such as KIM-1 in the use of early diagnosis, risk assessment, and disease monitoring these patients.

## 3. Cisplatin-Induced Nephrotoxicity

Of the total cardiac output, 25% is directed to the kidneys. As most drugs are renally cleared, they are the main site for drug-induced renal injury [57]. Unfortunately, despite nearly one quarter of AKI cases being related to drug nephrotoxicity, current assays are not able to predict patients at risk or to detect early stages of the disease [8,9]. The mechanism for drug-induced nephrotoxicity include tubular cell toxicity, crystal nephropathy, changes in glomerular hemodynamics, rhabdomyolysis, and thrombotic microangiopathy. However, detection in hospitalized patients is very often delayed by the lack of specific assays, while routine parameters such as sCr, BUN, and eGFR are influenced by several factors leading to erroneous mechanism identification and tardive diagnosis [58].

Cisplatin is a widely used anticancer drug especially in solid malignant tumors [59,60,61,62,63,64]. However, one of the main factors limiting the use of cisplatin (cis-diamminedichlorolatinum (II), CDDP) is the associated nephrotoxicity [65,66,67,68]. Frequently, toxic levels concentrating in the kidneys determine that injury occurs inside the proximal tubules, affecting to a lesser degree the glomeruli and the distal tubule. Interestingly, it seems that the S3 segment of the proximal tubules is affected even in the case of non-toxic drug concentration [16,69]. Increased levels determines tubulointerstitial disease with subsequent tubular necrosis, resulting in a sCr increase and GFR decrease [70]. 

Clinical studies illustrated that in 10 days of cisplatin therapy, sCr and urinary albumin increase while GFR decreases in 8–10% of patients [71]. Dose adjustments forced by these side effects can limit its clinical use and efficacy.

### 3.1. Cellular Mechanism 

Cisplatin associated renal injury first manifests through glutathione-S-transferase-mediated glutathione conjugates formation. Gamma-glutamyl transpeptidase (GGT) later cleaves the latter to cysteinyl-glycine derivates. These are converted by an aminopeptidase N (APN) into cysteine-conjugates that enter the proximal tubule epithelial cells. They are further metabolized to a reactive thiol group which is highly nephrotoxic [68]. The fact that GGT is predominantly found at the apical surface of the proximal tubule’s epithelial cells make it a desirable and possible target for managing cisplatin associated nephrotoxicity [72].

The mechanisms involve subsequent down-regulation of basolateral organic anion to kidney transporter-mediated uptake and cation transporters including organic cation transporter 2 (OCT2) and copper transporter1 (CTR1) [73,74,75,76]. Apically-localized efflux transporters such as antimicrobial extrusion protein (MATEs), multidrug resistance-associated proteins (MRPs) [77], and adenosine triphosphate (ATPases) mediate urinary cisplatin excretion [78].

Elevated proximal tubule cisplatin concentration leads to platinum complexes formation that activate AMP protein kinase (AMPK). This decreases autophagy, increases the DNA damage [79], kidney vascular resistance, tubular cells necrosis, and apoptosis and inflammation levels [80,81].

### 3.2. The Intrinsic and Extrinsic Pathway of Apoptosis

Vital in the process of apoptosis, caspases are initially inactive cysteine–aspartic proteases produced in response to a stimulus (intracellular/extracellular). The cleavage of the initiating caspase (i.e., caspase 8), followed by that of the executioner caspase (caspase 3) renders them active and determines DNA fragmentation with subsequent cell death [82]. 

Activation of at least one of the three possible pathways (apoptotic pathways) determines caspase 3 cleavage: It may be extrinsic through death receptors, intrinsic (mitochondrial pathway) and/or mediated via the endoplasmic reticulum (ER) [83].

Mitochondrial dysfunction is the main mechanism involved in the intrinsic apoptosis pathway while damage to the DNA material activates tumor suppressor gene *p53* [84]. p53 protein is involved in the development of cisplatin-induced nephrotoxicity because of its ability to inhibit the mitochondrial membrane bound anti-apoptotic proteins of the B-cell lymphoma (Bcl) group [85,86]. The latter are vital for both membrane structure maintenance and overall mitochondrial activity. As cisplatin levels increase, Bcl-2 is downregulated in response to p53 activation [87,88,89,90].

The extrinsic apoptosis pathway includes Fas ligand (FasL) and/or tumor necrosis factor alpha (TNFα)-mediated death receptor activation, that in turn stimulates intracellular caspases. Macrophages are the main source of TNFα. By binding to tumor necrosis factor receptor (TNFR), the latter initiates the apoptosis pathway of the intracellular caspases, resulting in cellular death [91,92]. Caspase 8 is the main initiator and it becomes active with cleavage. High-rate cleavage results in increased apoptosis and has been identified as a factor contributing to AKI exacerbation [93,94]. Another mechanism involves the presence of damage-associated molecular patterns (DAMPs), generated by intracellular injury. DAMPs stimulate toll-like receptor-4 (TLR-4), which in turn activates p38 mitogen protein kinase (MAPK) pathway, leading to increased TNF-α production in the kidney [95].

### 3.3. Oxidative Stress in Cisplatin-Induced AKI 

Another mechanism involved in cisplatin-nephrotoxicity is oxidative stress and the subsequent cytochrome P450 activation. In cisplatin-associated AKI, mitochondrial dysfunction associated oxidative stress leads to intracellular reactive oxygen species (ROS) accumulation. Previous research showed that cisplatin administration is followed by increased oxidative stress levels and alteration in the expression of various antioxidant enzymes [96,97,98]. Mitochondria is the home of the cellular ATP synthesis and the main source of ROS production. Cellular stress disrupts mitochondrial activity with increased ROS production and decreased ATP formation. ROS amplifies nicotinamide adenine dinucleotide phosphate (NADPH) and nicotinamide oxidase 2 (NOX-2) activity and alters glutathione peroxidase (GPX) and superoxide dismutase (SOD), reducing their antioxidant effect [99,100,101,102,103,104]. DNA damage results in ROS overproduction, with mitochondrial catalase (CAT), glutathione (GSH), and SOD inhibition and subsequent increase in renal tubular cells apoptosis [105,106,107]. Widely available techniques such as ELISA, immunoblotting, immunofluorescence, and immunohistochemistry may be used to measure the activity of the above-mentioned enzymes, taking into consideration that they may serve as a target in cisplatin induced nephrotoxicity. 

### 3.4. Inflammation Cytokines and Chemokines in Cellular Damage

Inflammation is a key mechanism in cisplatin associated AKI. Activated leukocytes and pro-inflammatory cytokines are required for tubular epithelial cells injury, initiating and prolonging the extent of the inflammation. NF-kB signaling pathway activation leads to increased secretion of TNF-α, IL-1, -6 through gene upregulation, thus contributing to AKI development and progression [108,109,110]. TNFα can be produced by injured renal tubules, leukocytes, macrophages, fibroblasts, and keratinocytes. 

By binding to the two available TNF receptors-1 and 2, respectively (TNFR1 and TNFR2), TNFα induces apoptosis by several pathways. It can activate NF-κB pathway, MAPK pathway or can determine cellular death through FasL and caspase 8 activation and subsequent cleavage [110].

**Figure 3:**
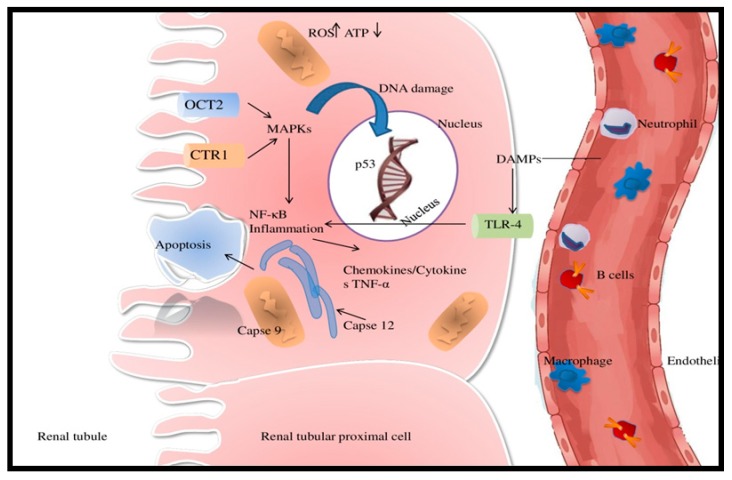
The complex mechanisms of cisplatin-induced acute kidney injury (AKI). Adenosine triphosphate (ATP); copper transporter 1 (Ctr1)(CTR1); damage-associated molecular pattern molecules (DAMPs); mitogen-activated protein kinase (MAPK); nuclear factor kappa-light-chain-enhancer of activated B cells (NF-κB); organic cation transporter (OCT2); reactive oxygen species (ROS); toll-like receptor-4 (TLR-4); tumor necrosis factor alpha (TNF-α).

Cisplatin induces numerous proximal tubular endothelial cells histological alterations, with subsequent changes in function. The inflammation is potentiated by the entrance of different immune cells inside epithelial cells. As such, mast cells, natural killer cells, macrophages, and lymphocytes (especially T) perpetuate the inflammatory process [111,112,113] (Figure 3).

Various pro-inflammatory cytokines and interleukins are secreted in the process, including IL-1, 1β, 6, 18, CXCL1, 8 and CCL2, 5, 10. Detecting them may be helpful in early AKI diagnosis in the context of cisplatin therapy. Moreover, they may hold better sensitivity and specificity than current assays. 

Activated CD4+ T lymphocytes contribute to the apoptosis signaling pathway by secreting and expressing different molecules, including KIM-1 (plays a crucial role in phagocytosis), T cell immunoglobulin mucin (TIM-1), Hcvr1, and FasL (death activator receptor) [114,115]. The mechanisms behind cisplatin nephrotoxicity are complex, and since there is so far no effective therapy for cisplatin-induced AKI, early detection and subsequent prevention are crucial. This is why newer biomarkers such as KIM-1 are needed.

## 4. Assessment of KIM-1 in Cisplatin-Induced Nephrotoxicity

Newer biomarkers with increased sensitivity and specificity are needed to ensure early AKI detection, and even provide us with newer therapeutic targets. Not only this, but an ideal AKI biomarker would enable renal function monitoring during treatment. Several authors have shown that decreased concentrations of some AKI biomarkers are associated with recovery [116,117]. Therefore, it is necessary to study renal biomarkers with possible predictive values in regard to nephrotoxicity and AKI development that can ensure both early diagnosis and therapeutic interventions, thus improving overall prognosis [118].

In an experimental study, KIM-1 was the most specific urinary biomarker in detecting AKI [8]. Both animal and human studies revealed KIM-1 as sensitive in detecting cisplatin associated nephrotoxicity [119,120,121]. KIM-1 levels increased after one-day post-cisplatin administration and this elevation correlated with AKI. Moreover, a form of kidney injury was reported in more than one quarter of patients after the first dose of cisplatin [122,123]. Authors concluded that this biomarker may be able to predict cisplatin-induced AKI [124]. In a prospective study including 123 patients under platinum chemotherapeutics, urinary levels of KIM-1, NGAL, and cystatin C showed statistically significant elevation at day three after treatment initiation in AKI patients. Importantly, it seems that KIM-1 elevations precede sCr rise [125]. A different study showed that high-mobility group box protein 1 (HMGB1), NGAL, and KIM-1 increased significantly one day after a 10 μM dose of cisplatin [126]. HMGB1 showed a more rapid and significant increase, of nearly seven times higher at six hours, compared to KIM-1, that nearly doubled its levels after 24 h.

Lung-cancer patients under cisplatin therapy showed increased KIM-1 and monocyte chemotactic protein-1 (MCP-1) levels and lower NGAL, β2-microglobulin, and NAG. The reported AUC for the two biomarkers was 0.858 and 0.850, respectively, showing an increased predictive ability for AKI detection [127]. 

A Canadian prospective study included pediatric oncologic patients at risk for renal damage and/or thrombo-embolic events during a three yeare period. One of the study’s goals was analyzing the ability of KIM-1 and NGAL in early AKI detection and their utility in identifying patients at risk for long-term complications such as CKD and associated secondary hypertension. Taking into consideration that KIM-1 is specific for proximal tubular injuries, as opposed to NGAL which increases in both proximal and distal lesions, combining the two may ensure early diagnosis [122].

In a study conducted by Salem et al. KIM-1, caspase 3, and intercellular adhesion molecule-1 ICAM-1 increased significantly after cisplatin administration [128]. In addition, both KIM-1 and NGAL were elevated in cisplatin-treated subjects [129]. Doxorubicin and cisplatin treated specimens exhibited elevated KIM-1, NGAL, and human macrophage colony-stimulating factor (M-CSF) levels [130]. 

More so, cisplatin administration induced proximal tubule histological lesions, with a subsequent increase in several biomarkers, from the conventional sCr and BUN to the newer NGAL and KIM-1 [131]. Elevated urinary KIM-1 levels were found in both young and aged rats three days after cisplatin exposure [132,133]. 

In a prospective study, patients treated with first-line chemotherapy showed increased KIM-1, sCr, and clusterin three days after treatment initiation. On the contrary, rats treated with intraperitoneal dexamethasone and/or cisplatin showed increased plasma cystatin C (pCysC) levels without, however overt histological damage or other biomarker elevation [134]. McDuffie et al. [135] showed that animals receiving a 0.75 mg/kg daily dose of cisplatin for five days, showed a discrete elevation of KIM-1 in only two specimens. However, histological examination revealed both medullar and cortical tubular epithelium lesions despite absent urinary KIM-1 elevation. In the two specimens, elevated KIM-1, IL-1B, and various chemokines (CCL 2, CCL20, CXC motif ligand 1, 10) were identified in proximal tubule epithelial cells. It follows that urinary KIM-1 and CCL2 could show promise as novel and specific biomarkers in detecting proximal tubular lesions [136].

Interestingly, in a 3D bioengineered kidney tissue model, KIM-1 and NGAL showed increased sensitivity in revealing both acute and chronic cisplatin or doxorubicin associated renal toxicity cultures [137].

KIM-1 is a novel biomarker that seems to be more sensitive and specific in identifying and monitoring nephrotoxic associated AKI. Moreover, its levels closely mirror the amount of renal tissue damage. Using such biomarkers in addition to classical assays such as sCr in routine clinical practice will allow better risk stratification and overall management, based on early diagnosis and appropriate intervention. However, further studies are warranted to validate its utility and identify proper cut-off values in early toxic renal injury.

## 5. Future Directions/Perspective Regarding KIM-1 in Drug-Induced Nephrotoxicity

KIM-1 is used in both preclinical and clinical studies to identify and monitor drug-induced kidney injury, therefore rapid urinary tests are currently under development. Several studies showed the role of specific biomarkers in diagnosis and follow-up of AKI patients. Among these, KIM-1 also has a possible predictive role in different pathologies alone or associated with other biomarkers. This biomarker was approved by the FDA more than a decade ago, as an nephrotoxic biomarker for different drugs in use [138]. Research teams continue to investigate the nephrotoxic mechanisms of different widely used chemotherapy drugs, with hopes that in the future a panel of specific renal biomarkers can be used as the gold standard for early renal injury detection. Previous studies investigated the effect of the most frequent chemotherapy agents used in clinical practice on tubular renal cells and the effect on renal biomarkers [139,140,141,142,143,144,145,146,147] (Table 1).

KIM-1 dipsticks measurements were developed using microbe-based assays. The band’s intensity correlated with histopathological and immunohistochemical-identified lesions. In post-operative cisplatin administration AKI patients, the KIM-1 dipstick band was positive [148]. Recent studies reported the development of two other quantitative KIM-1 measurements. The first one was represented by a microbead-based KIM-1 ELISA, while the second one was a laminar flow dipstick assay.

Both methods confirmed that KIM-1 levels were significantly more elevated as compared to sCr, BUN, and NAG after just 10 minutes of renal ischemia [149]. We hope that future studies can provide enough evidence to promote the use of KIM-1 dipsticks for rapid diagnostic assay in early kidney injury. Interestingly anti TIM/KIM-1 antibodies can protect against future injury in renal cells, thus preventing renal disease. Authors showed that anti-TIM-1 antibodies RMT1-10 had protective effects in mice against crescentic and proliferative glomerulonephritis through their effects on Th1 and Th17 lymphocytes [150]. 

In addition, KIM-1 can also be regarded as a potential therapeutic target in clear cell renal carcinoma, as it shown to inhibit 786-0 cell growth both in vitro and in vivo [151]. Through immunological mechanisms, there is a possibility that in the future new therapeutic agents are discovered for nephrotoxic induced-AKI.

## 6. Strategies to Prevent Cisplatin Nephrotoxicity

Cisplatin is a widely used chemotherapeutic agent for a broad spectrum of malignancies. However, renal injury following its administration limits its use. There are several established risk factors for developing cisplatin induced AKI, including previous cisplatin therapy, increased dose regimens, preexisting renal disease and combination of cisplatin with another nephrotoxic agent. Therefore, research focuses on developing more efficient preventive measures. One strategy includes lower cisplatin dose regimens with intensive intravenous hydration with saline infusion and concomitant mannitol administration (also administered after cisplatin infusion ended). However, it seems that normal saline with mannitol is inferior to saline alone or in combination with furosemide in cisplatin associated acute renal failure. Despite not being able to influence cisplatin cellular accumulation, normal saline alters cellular sensitivity to the chemotherapeutic agent [152]. Magnesium has also proven to be effective in combination with proper hydrating regimens [153].

Recent studies focused on efficient herbal natural antioxidants, as they may partially prevent or modulate the level of oxidative stress and subsequent inflammation that characterize cisplatin administration [154]. Selenium and vitamin E are known antioxidants and have been shown to reduce oxidative stress following cisplatin therapy [155]. Other known antioxidants such as dimethyl thiourea (DMTU), vitamin C, and alpha-lipoic acid have renal protective effects during cisplatin therapy [156,157]. In vitro studies showed that urinary KIM-1, NGAL, and HMGB levels have been reduced by *Nelumbo nymphaea* administration in vivo [158]. Black seed/black cumin (*Nigella sativa*) has also proven to be protective in this setting [127]. Hosseinian et al. administered it for a week and compared its effects with vitamin E infusion in preventing cisplatin induced AKI [159]. The authors concluded that *Nigella sativa* managed to reduce cisplatin renal toxic effects as compared to vitamin E and that these protective effects were dose-dependent. The beneficial effects of antioxidant therapy can be explained by the pathophysiological importance of ROS and subsequent mitochondrial dysfunction in the nephrotoxic effects of cisplatin [160].

Another explored strategy is combining a xanthine-oxidase inhibitor (allopurinol) with a glutathione peroxidase inhibitor (ebselen) [161]. This combination has been shown to reduce ROS generation. Similarly, the addition of an organic thiophosphate (amifostine) in ovarian/non-small cell lung carcinoma cisplatin treated patients seems to limit free radical production with subsequent protective effects [162]. Although there is not a clearly established role, N-acetylcysteine and theophylline may also be used in this setting [163].

Continuous intravenous infusion of cimetidine (OCT2-inhibitor) efficiently prevented cisplatin-nephrotoxicity without influencing its antineoplastic effects [164]. For their ability to limit glutathione-cisplatin derivates formation, thiol agents are also regarded as potential protective agents. FDA approved Amfifosten for AKI-prevention in cisplatin treated patients with either non-small cell lung carcinoma or advanced staged ovarian cancer [165]. In cisplatin treated animal studies, levosimendan managed to reduce urinary albumin/creatinine ratio, as well as KIM-1 and NAG levels [142,166]. Similarly, in Fluorouracil (5-FU) treated patients, camel milk can limit the associated nephrotoxic effects [143]. 

Expanding the effects of p53 manipulation beyond cisplatin associated nephrotoxicity, administration of p53 antagonists could enhance chemotherapeutics effects by malignant cellular sensitization, as p53 seems to be responsible for antineoplastic drug resistance [141]. 

Selectively inhibiting p53 is a tempting field of research as a possible therapeutic target in managing AKI in cisplatin treated patients.

Despite several agents being currently regarded as having possible protective effects against cisplatin-associated AKI, this complication is still difficult to predict, to diagnose early and properly manage, as the nephrotoxic effects must be weighed against the tumor extension. Newer strategies are under development in the attempt to limit cisplatin nephrotoxic effects without altering its properties as a potent chemotherapeutic agent. 

## 7. Discussion

Taking into consideration that AKI remains a global issue and that its etiology varies, more studies investigate its mechanisms, early detection, preventive, and therapeutic strategies. Neoplasia is growing in incidence; however each and every chemotherapeutic agent has several adverse effects, including nephrotoxicity. Using more specific assays in identifying chemotherapeutics’ toxic effects enables researchers to better monitor these effects and thoroughly evaluate renal structure and function alterations. Cisplatin (Platinol) a platinum-based chemotherapy drug is used frequently in many types of neoplasia.

Cisplatin-induced nephrotoxicity leads to dose and time-dependent molecular changes. 

Research currently focuses on various models of cisplatin associated AKI. Among known mechanisms are mitochondrial dysfunction with subsequent ROS formation and inflammation, apoptotic pathways, and autophagy. In preventing and/or limiting the extent of cisplatin induced AKI, adequate hydration is still the cornerstone.

Taking into consideration that current assays lack sensitivity and specificity and that they are elevated only late in the progress of the disease, newer, more sensitive and specific for proximal tubular injury biomarkers are required in cisplatin induced AKI. Of note, before administering any potential nephrotoxic drug, a complete evaluation of renal function should be made, with close follow-up of high-risk patients. Properly identifying and early diagnosis of cisplatin induced nephrotoxicity becomes all the more important since most patients recover upon drug interruption and with appropriate therapeutic measures. 

The prediction ability of novel biomarkers still remains uncertain, however their utility in early diagnosis is of outmost importance. KIM-1 was isolated in atrophic epithelial cells of the proximal tubule; this biomarker shows promise in early diagnosis of renal damage. Its soluble form is a 90 kDa molecule (as compared to the membrane bound 104 kDa form) found in the urine of both animals and humans with renal injury. Its presence was associated with AKI in several experimental studies [34,41,42,43,44,45,46,47,48,49,50] and some authors suggest that it may be predictive of cisplatin nephrotoxicity [119,120,121,122,123,124,125,126,127,128,129,130,131,132,133,134,135]. Anti-KIM-1 antibodies have been developed as a potential therapy in neoplasia characterized by KIM-1 overexpression (renal, ovarian, and lung carcinomas) [167,168]. 

Future studies are trying to validate newer, more specific biomarkers in clinical practice for early diagnosis, monitoring, and even novel therapeutic targets in cisplatin induced AKI.

## 8. Conclusions

It is anticipated that early detection AKI biomarkers would be available for use in laboratory research and clinical studies relatively soon. The development of rapid, dipstick assays for KIM-1 could enable a better cisplatin associated AKI diagnosis, also improving patients’ care. As identifying both high-risk patients and incipient AKI are both desiderates of these new biomarkers, if proven to be efficient, these assays will become useful even in emergency settings. Urinary KIM-1 levels are elevated in cisplatin induced AKI and may help in the differential diagnosis of proximal epithelial cells injuries. Although further studies are needed to explore the mechanisms behind cisplatin nephrotoxicity and the role of KIM-1 in this setting, this novel biomarker is showing promise in early diagnosis and prognosis of AKI.

## Figures and Tables

**Table 1:** Neoplastic drugs and their effect on renal biomarkers.

Chemotherapy Agents	Therapeutic Doses	Administration Time and Detection	Increased Detection of Serum/Urine/Immunostaining	References
**Cisplatin (Cl_2_H_6_N_2_P)**	Dose ranging from 0.01 mM to 100 mM	KIM-1 detection after 2 weeks	↑ KIM-1, ↑NGAL	[137]
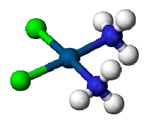	6 mg/kg	3rd day	↑ KIM-1, clusterin	[24]
	1 mg/kg/day	3rd day	↑ KIM-1, clusterin	[139]
	6 mg/kg (1 mg/mL)	3rd day	↑ KIM-1, clusterin, plasma cystatin	[133]
	One dose: 6 mg/kg	7th day and 10th day	↑ NGAL ↑ urinary NAG, ↑ KIM-1	[141]
	50 mg/m^2^	2nd and 3rd day	↑ KIM-1, NGAL and cystatin	[125]
	100 µmol/L	3rd day	↑ KIM-1, ↑NGAL	[130]
	5 μM	24 h	↑ KIM-1, ↑NGAL	[129]
	5 mg/kg	2nd day	↑KIM-1	[136]
	400 μM during 6 h or 10 μM during 1 day	6, 24, 48 h	↑ NGAL, ↑KIM-1, ↑HMGB1	[126]
	80 mg/m^2^, 125 mg/body	7th day	↑ KIM-1, ↑ MCP-1 ↑ NGAL	[127]
**Doxorubicin (C_27_H_29_NO_11_)**	0.001 mM to 0.2 mM	0, 3, 7, 10, 14 day	↑KIM-1 ↑NGAL	[137]
	20 mg/kg	20th day	↑ KIM-1	[145]
**Fluorouracil (5-FU) (C_4_H_3_FN_2_O_2_)**	50 mg/kg/day	17th–21st day	↑ KIM-1, ↑ NGAL	[140]
	150 mg/kg	21st day	↑ KIM-1	[147]

## Abbreviations

CKD	chronic kidney disease
AKI	acute kidney injury
sCr	serum creatinine
GFR	glomerular filtration rate
eGFR	estimated glomerular filtration rate
BUN	blood urea nitrogen
KIM-1	kidney injury molecule-1
ATN	acute tubular nephritis
OCTs	organic cation transporters
CTRs	copper transporters
ATP	adenosine triphosphate
AMPK	AMP-activated protein kinase
ER	endoplasmic reticulum
Bcl	B-cell lymphoma
FasL	Fas ligand
TNFα	tumor necrosis factor alpha
TNFR	tumor necrosis factor receptor
MAPK	mitogen-activated protein kinase
NADPH	nicotinamide adenine dinucleotide phosphate
DAMPs	damage-associated molecular pattern molecules
TLR-4	toll-like receptor-4
ROS	reactive oxygen species
NOX2	NADPH oxidase 2
GPX	glutathione peroxidase
SOD	superoxide dismutase
CAT	mitochondria inhibits catalase
TNFR	TNF receptor
JNK	c-Jun N-terminal kinase
ERK	extracellular signal–regulated kinases
Tim-1	T cell immunoglobulin mucin-1
NAG	N-acetyl-β-glucosaminidase
DMTU	Dimethylthiourea

## References

[1] World Health Organization (2013). Global Action Plan. for the Prevention and Control. of Noncommunicable Diseases, 2013–2020.

[2] Mehta R.L., Cerdá J., Burdmann E.A., Tonelli M., García-García G., Jha V., Susantitaphong P., Rocco M., Vanholder R., Sever M.S. (2015). International Society of Nephrology’s 0by25 initiative for acute kidney injury (zero preventable deaths by 2025): A human rights case for nephrology. Lancet.

[3] Faught L.N., Greff M.J., Rieder M., Koren G. (2014). Drug-induced acute kidney injury in children. Br. J. Clin. Pharmacol..

[4] Sinert R., Peacock P.R., Tintinalli J.E., Stapczynski J.S., Ma A.J., Yealy D.M., Mecklern G.D., Cline D.M. (2016). Acute kidney injury. Tintinalli’s Emergency Medicine.

[5] Perazella M.A. (2018). Pharmacology behind common drug nephrotoxicities. Clin. J. Am. Soc. Nephrol..

[6] Dhodi D.K., Bhagat S.B., Pathak D., Patel S.B., Dinesh K. (2014). Drug-induced nephrotoxicity. Int. J. Basic Clin. Pharmacol..

[7] Loghman-Adham M., Kiu Weber C.I., Ciorciaro C., Mann J., Meier M. (2012). Detection and management of nephrotoxicity during drug development. Expert Opin. Drug Saf..

[8] Bonventre J.V., Vaidya V.S., Schmouder R., Feig P., Dieterle F. (2010). Next-generation biomarkers for detecting kidney toxicity. Nat. Biotechnol..

[9] McCullough P.A., Bouchard J., Waikar S.S., Siew E.D., Endre Z.H., Goldstein S.L., Koyner J.L., Macedo E., Di Somma S. (2013). Implementation of novel biomarkers in the diagnosis, prognosis, and management of acute kidney Injury: Executive summary from the tenth consensus conference of the acute dialysis quality initiative (ADQI). ADQI Consensus on AKI Biomarkers and Cardiorenal Syndromes.

[10] Sari Z. (2019). Nephrotoxic Effects of Drugs Poisoning in the Modern World. Ren. Fail..

[11] Paueksakon P., Fogo A.B. (2017). Druginduced nephropathies. Histopathology.

[12] Kellum J.A., Lameire N., Aspelin P., Barsoum R.S., Burdmann E.A., Goldstein S.L., Herzog C.A., Joannidis M., Kribben A., Levey A.S. (2012). Kidney Disease: Improving Global Outcomes (KDIGO) Acute Kidney Injury Work Group. KDIGO clinical practice guideline for acute kidney injury. Kidney Int. Suppl..

[13] Murray P.T., Mehta R.L., Shaw A., Ronco C., Endre Z., Kellum J.A., Chawla L.S., Cruz D., Ince C., Okusa M.D. (2014). Potential use of biomarkers in acute kidney injury: Report and summary of recommendations from the 10th Acute Dialysis Quality Initiative consensus conference. Kidney Int..

[14] Nagai J., Takano M. (2010). Molecular-targeted approaches to reduce renal accumulation of nephrotoxic drugs. Expert Opin. Drug Metab. Toxicol..

[15] Kim S.Y., Moon A. (2012). Drug-Induced Nephrotoxicity and Its Biomarkers. Biomol. Ther.

[16] Ozkok A., Edelstein C.L. (2014). Pathophysiology of cisplatin-induced acute kidney injury. BioMed Res. Int..

[17] Labaye J., Sarret D., Duvic C., Hérody M., Didelot F., Nédélec G., Noël L.H. (2005). Renal toxicity of oxaliplatin. Nephrol. Dial. Transplant..

[18] Ulusakarya A., Misra S., Haydar M., Habert H., Castagne V., Gumus Y., Delmas-Marsalet B., Machover D. (2010). Acute renal failure related to oxaliplatin-induced intravascular hemolysis. Med. Oncol..

[19] Yaghobi Joybari A., Sarbaz S., Azadeh P., Mirafsharieh S.A., Rahbari A., Farasatinasab M., Mokhtari M. (2014). Oxaliplatin-induced renal tubular vacuolization. Ann. Pharmacother..

[20] Isnard-Bagnis C., Launay-Vacher V., Karie S., Deray G., De Broe M., Porter G., Bennett W., Deray G. (2008). Anticancer drugs. Clinical Nephrotoxins Renal Injury from Drug and Chemicals.

[21] Feigelstock D., Thompson P., Mattoo P., Kaplan G.G. (1998). The human homolog of HAVcr-1 codes for a hepatitis A virus cellular receptor. J. Virol..

[22] Chiusolo A., Defazio R., Zanetti E., Mongillo M., Mori N., Cristofori P., Trevisan A. (2010). Kidney injury molecule-1 expression in rat proximal tubule after treatment with segment-specific nephrotoxicants: A tool for early screening of potential kidney toxicity. Toxicol. Pathol..

[23] Vaidya V.S., Ozer J.S., Dieterle F., Collings F.B., Ramirez V., Troth S. (2010). Kidney injury molecule-1 outperforms traditional biomarkers of kidney injury in preclinical biomarker qualification studies. Nat. Biotechnol..

[24] Sasaki D., Yamada A., Umeno H. (2011). Comparison of the course of biomarker changes and kidney injury in a rat model of drug-induced acute kidney injury. Biomarkers.

[25] [Internet] FDA. http://www.fda.gov/bbs/topics/NEWS/2008/NEW01850.html.

[26] Dieterle F., Sistare F., Goodsaid M., Papaluca J.S., Ozer C.P., Webb W., Baer A., Senagore M.J., Walker E., Sultana S. (2010). Renal biomarker qualification submission: A dialog between the FDA-EMEA and Predictive Safety Testing Consortium. Nat. Biotechnol..

[27] Harpur E., Ennulat D., Hoffman D., Betton G., Gautier J.C., Riefke B., Bounous D., Schuster K., Beushausen S., Guffroy M. (2011). Nephrotoxicity. Toxicol. Sci..

[28] Ichimura T., Bonventre J.V., Bailly V., Wei H., Hession C.A., Cate R.L., Sanicola M. (1998). Kidney injury molecule-1 (KIM-1), a putative epithelial cell adhesion molecule containing a novel immunoglobulin domain, is up-regulated in renal cells after injury. J. Biol. Chem..

[29] Hubank M., Schatz D.G. (1994). Identifying differences in mRNA expression by representational difference analysis of cDNA. Nucl. Acids Res..

[30] Kuchroo V.K., Meyers J.H., Umetsu D.T., DeKruyff R.H. (2006). TIM family of genes in immunity and tolerance. Adv. Immunol..

[31] Bonventre J.V. (2009). Kidney injury molecule-1 (KIM-1), “A urinary biomarker and much more”. Nephrol. Dial. Transplant..

[32] Van Timmeren M.M., van den Heuvel M.C., Bailly V., Bakker S.J., van Goor H., Stegeman C.A. (2007). Tubular kidney injury molecule-1 (KIM-1) in human renal disease. J. Pathol..

[33] Bailly V., Zhang Z., Meier W., Cate R., Sanicola M., Bonventre J.V. (2002). Shedding of kidney injury molecule-1, a putative adhesion protein involved in renal regeneration. J. Biol. Chem..

[34] Lim A.I., Tang S.C., Lai K.N., Leung J.C. (2013). Kidney injury molecule-1: More than just an injury marker of tubular epithelial cells?. J. Cell. Physiol..

[35] Visnagri A., Kandhare A.D., Bodhankar S.L. (2015). Renoprotectiveeffect of berberine via intonation on apoptosis andmitochondrial-dependent pathway in renal ischemia reperfusion-induced mutilation. Ren. Fail..

[36] Bonventre J.V., Yang L. (2010). Kidney injury molecule-1. Curr. Opin. Crit. Care.

[37] Brooks C.R., Bonventre J.V. (2015). KIM-1/TIM-1 in proximal tubular cell immune response. Oncotarget.

[38] Brooks C.R., Yeung M.Y., Brooks Y.S., Chen H., Ichimura T., Henderson J.M., Bonventre J.V. (2015). KIM-1-/TIM-1-mediated phagocytosis links ATG5-/ULK1-dependent clearance of apoptotic cells to antigen presentation. EMBO J..

[39] Tsigou E., Psallida V., Demponeras C., Boutzouka E., Baltopoulos G. (2013). Role of new biomarkers: Functional andstructural damage. Crit. Care Res. Pract..

[40] Ichimura T., Brooks C.R., Bonventre J.V. (2012). Kim-1/ Tim-1 and immune cells: Shifting sands. Kidney Int..

[41] Gobe G.C., Coombes J.S., Fassett R.G., Endre Z.H. (2015). Biomarkers of drug-induced acute kidney injury in the adult. Expert Opin. Drug Metab. Toxicol..

[42] Kadioglu T., Uzunlulu M., Yigit Kaya S., Oguz A., Gonenli G., Isbilen B., Isman F.K. (2016). Urinary kidney injury molecule-1 levels as a marker of early kidney injury in hypertensive patients. Minerva Urol. Nefrol..

[43] Sabbisetti V.S., Waikar S.S., Antoine D.J., Smiles A., Wang C., Ravisankar A., Ito K., Sharma S., Ramadesikan S., Lee M. (2014). Blood kidney injury molecule-1 is a biomarker of acute and chronic kidney injury and predicts progression to ESRD in type I diabetes. J. Am. Soc. Nephrol..

[44] Perco P., Oberbauer R. (2008). Kidney injury molecule-1 as a biomarker of acute kidney injury in renal transplant recipients. Nat. Clin. Pract. Nephrol..

[45] Medic B., Rovcanin B., Basta Jovanovic G., Radojevic-Skodric S., Prostran M. (2015). Kidney injury molecule-1 and cardiovascular diseases: From basic science to clinical practice. BioMed Res. Int..

[46] Shao X., Tian L., Xu W., Zhang Z., Wang C., Qi C., Ni Z., Mou S. (2014). Diagnostic Value of Urinary Kidney Injury Molecule 1 for Acute Kidney Injury: A Meta-Analysis. PLoS ONE.

[47] Schulz C.A., Engström G., Nilsson J., Almgren P., Petkovic M., Christensson A., Nilsson P.M., Melander O., Melander M.O. (2019). Plasma kidney injury molecule-1 (p-KIM-1) levels and deterioration of kidney function over 16 years. Nephrol. Dial. Transplant..

[48] John M., Arthur E.G., Hill J.L., Lewis E.C., Neely B.A., Janech M.G., Tumlin J.A., Chawla L.S., Shaw A.D. (2014). Evaluation of 32 urine biomarkers to predict the progression of acute kidney injury after cardiac surgery. Kidney Int..

[49] Pinches M.D., Betts C.J., Bickerton S.J., Beattie L., Burdett L.D., Thomas H.T., Derbyshire N.A., Moores M., Price S.A. (2012). Evaluation of novel urinary renal biomarkers: Bio- logical variation and reference change values. Toxicol. Pathol..

[50] Tsuji S., Sugiura M., Tsutsumi S., Yamada H. (2017). Sex differences in the excretion levels of traditional and novel urinary biomarkers of nephrotoxicity in rats. J. Toxicol. Sci..

[51] Krawczeski C.D., Goldstein S.L., Woo J.G., Wang Y., Piyaphanee N., Ma Q., Bennett M., Devarajan P. (2011). Temporal relationship and predictive value of urinary acute kidney injury biomarkers after pediatric cardiopulmonary bypass. J. Am. Coll. Cardiol..

[52] Sun I.O., Shin S.H., Cho A.Y., Yoon H.J., Chang M.Y., Lee K.Y. (2017). Clinical significance of NGAL and KIM-1 for acute kidney injury in patients with scrub typhus. PLoS ONE.

[53] Ornellas F.M., Ornellas D.S., Martini S.V. (2017). Bone Marrow–Derived Mononuclear Cell Therapy Accelerates Renal Ischemia- Reperfusion Injury Recovery by Apoptotic Related Molecules. Cell. Physiol. Biochem..

[54] Szeto C.C., Kwan B.C., Lai K.B., Lai F.M., Chow K.M., Wang G., Luk C.C., Li P.K. (2010). Urinary expression of kidney injury markers in renal transplant recipients. Clin. J. Am. Soc. Nephrol..

[55] Nielsen S.E., Schjoedt K.J., Astrup A.S., Tarnow L., Lajer M., Hansen P.R., Parving H.H., Rossing P. (2010). Neutrophil gelatinase-associated lipocalin (ngal) and kidney injury molecule 1 (kim1) in patients with diabetic nephropathy: A cross-sectional study and the effects of lisinopril. Diabet. Med..

[56] Zhang P.L., Mashni J.W., Sabbisetti V.S., Schworer C.M., Wilson G.D., Wolforth S.C., Kernen K.M., Seifman D.B., Amin B.M., Geddes T.J. (2014). Urine kidney injury molecule-1: A potential non-invasive biomarker for patients with renal cell carcinoma. Int. Urol. Nephrol..

[57] Fuchs T.C., Hewitt P. (2011). Biomarkers for drug-induced renal damage and nephrotoxicity-an overview for applied toxicology. AAPS J..

[58] Van Meer L., Moerland M., Cohen A.F., Burggraaf J. (2014). Urinary kidney biomarkers for early detection of nephrotoxicity in clinical drug development. Br. J. Clin. Pharmacol..

[59] Vokes E.E. (2010). Induction chemotherapy for head and neck cancer: Recent data. Oncologist.

[60] Ismaili N., Amzerin M., Flechon A. (2011). Chemotherapy in advanced bladder cancer: Current status and future. J. Hematol. Oncol..

[61] Moxley K.M., McMeekin D.S. (2010). Endometrial carcinoma: A review of chemotherapy, drug resistance, and the search for new agents. Oncologist.

[62] Gronwald J., Byrski T., Lubinski J., Narod S.A. (2012). Cisplatin in breast cancer treatment in BRCA1 carriers. Hered Cancer Clin. Pract..

[63] Zarogoulidis K., Zarogoulidis P., Darwiche K., Boutsikou E., Machairiotis N., Tsakiridis K., Katsikogiannis N., Kougioumtzi I., Karapantzos I., Huang H. (2013). Treatment of non-small cell lung cancer (NSCLC). J. Thorac. Dis..

[64] Chan B.A., Coward J.I.G. (2013). Chemotherapy advances in small-cell lung cancer. J. Thorac. Dis..

[65] Tucker B.M., Perazella M.A., Lerma E.V., Sparks M.A., Topf J. (2018). Medications. Nephrology Secrets.

[66] Perazella M.A. (2012). Onco-nephrology: Renal toxicities of chemotherapeutic agents. Clin. J. Am. Soc. Nephrol..

[67] Dos Santos N.A., Carvalho Rodrigues M.A., Martins N.M., dos Santos A.C. (2012). Cisplatininduced nephrotoxicity and targets of nephroprotection: An update. Arch. Toxicol..

[68] Miller R.P., Tadagavadi R.K., Ramesh G., Reeves W.B. (2010). Mechanisms of cisplatin nephrotoxicity. Toxins.

[69] Bolisetty S., Traylor A., Joseph R., Zarjou A., Agarwal A. (2016). Proximal tubule-targeted heme oxygenase-1 in cisplatin-induced acute kidney injury. Am. J. Physiol. Ren. Physiol..

[70] Hughes P.J. (2017). Pathophysiologic Mechanisms of Selected Types of Nephrotoxicity. https://emedicine.medscape.com/article/1925868.

[71] Atilano-Roque A., Wen X., Aleksunes L.M., Joy M.S. (2016). Nrf2 activators as potential modulators of injury in human kidney cell. Toxicol. Rep..

[72] Fliedl L., Wieser M., Manhart G., Gerstl M.P., Khan A., Grillari J., Grillari-Voglauer R. (2014). Controversial role of gamma-glutamyl transferase activity in cisplatin nephrotoxicity. ALTEX.

[73] Ward P.D., La D., McDuffie J.E., Gowder S. (2013). Renal transporters and biomarkers in safety assessment. New Insights into Toxicity and Drug Testing.

[74] Ciarimboli G. (2014). Membrane transporters as mediators of cisplatin side effects. Anticancer Res..

[75] Saito Y., Okamoto K., Kobayashi M., Narumi K., Furugen A., Yamada T., Iseki K. (2017). Magnesium co-administration decreases cisplatin-induced nephrotoxicity in the multiple cisplatin administration. Life Sci..

[76] Ciarimboli G. (2012). Membrane Transporters as Mediators of Cisplatin Effects and Side Effects. Scientifica.

[77] Estrela G.R., Wasinski F., Felizardo R.J.F., Souza L.L., Câmara N.O.S., Bader M., Araujo R.C. (2017). MATE-1 modulation by kinin B1 receptor enhances cisplatin efflux from renal cells. Mol. Cell. Biochem..

[78] Harrach S., Ciarimboli G. (2015). Role of transporters in the distribution of platinum-based drugs. Front. Pharmacol..

[79] Zhu N., Pabla C., Tang C., He L., Dong Z. (2015). DNA damage response in cisplatin-induced nephrotoxicity. Arch. Toxicol..

[80] Manohar S., Leung N. (2018). Cisplatin nephrotoxicity: A review of the literature. J. Nephrol..

[81] Xu Y., Ma H., Shao J., Wu J., Zhou L., Zhang Z., Wang Y., Huang Z., Ren J., Liu S. (2015). A role for Tubular Necroptosis in Cisplatin-Induced AKI. JASN.

[82] Basu A., Krishnamurthy S. (2010). Cellular responses to Cisplatin-induced DNA damage. J. Nucl. Acids.

[83] Sharp C.N., Doll M.A., Dupre T.V., Shah P.P., Subathra M., Siow D., Arteel G.E., Megyesi J., Beverly L.J., Siskind L.J. (2016). Repeated administration of low-dose cisplatin in mice induces fibrosis. Am. J. Physiol. Renal. Physiol..

[84] Vaseva A.V., Moll U.M. (2009). The mitochondrial p53 pathway. Biochim. Biophys. Acta.

[85] Siskind L.J. (2016). Repeated administration of low-dose cisplatin in mice induces fibrosis. Am. J. Physiol. Ren. Physiol..

[86] Bhatt K., Zhou L., Mi Q.S., Huang S., She J.X., Dong Z. (2010). MicroRNA-34a is induced via p53 during cisplatin nephrotoxicity and contributes to cell survival. Mol. Med..

[87] Nakagawa T., Kakizoe Y., Iwata Y., Miyasato Y., Mizumoto T., Adachi M., Izumi Y., Kuwabara T., Suenaga N., Narita Y. (2018). Doxycycline attenuates cisplatin-induced acute kidney injury through pleiotropic e_ects. Am. J. Physiol. Ren. Physiol..

[88] Watanabe M., Oe Y., Sato E., Sekimoto A., Sato H., Ito S., Takahashi N. (2019). Protease-activated receptor 2 exacerbates cisplatin-induced nephrotoxicity. Am. J. Physiol. Ren. Physiol..

[89] Soni H., Matthews A.T., Pallikkuth S., Gangaraju R., Adebiyi A. (2019). Gamma-secretase inhibitor DAPT mitigates cisplatin-induced acute kidney injury by suppressing Notch1 signaling. J. Cell. Mol. Med..

[90] Zhang W., Hou J., Yan X., Leng J., Li R., Zhang J., Xing J., Chen C., Wang Z., Li W. (2018). Platycodon grandiflorum Saponins Ameliorate Cisplatin-Induced Acute Nephrotoxicity through the NF-kappaB-Mediated Inflammation and PI3K/Akt/Apoptosis Signaling Pathways. Nutrients.

[91] Sen Z., Jie M., Jingzhi Y., Dongjie W., Dongming Z., Xiaoguang C. (2017). Total Coumarins from Hydrangea paniculata Protect against Cisplatin-Induced Acute Kidney Damage in Mice by Suppressing Renal Inflammation and Apoptosis. Evid. Based Complement. Altern. Med..

[92] Parameswaran N., Patial S. (2010). Tumor necrosis factor-alpha signaling in macrophages. Crit. Rev. Eukaryot. Gene Expr..

[93] Dupre T.V., Doll M.A., Shah P.P., Sharp C.N., Kiefer A., Scherzer M.T., Saurabh K., Saforo D., Siow D., Casson L. (2016). Suramin protects from cisplatin-induced acute kidney injury. Am. J. Physiol. Ren. Physiol..

[94] Dupre T.V., Doll M.A., Shah P.P., Sharp C.N., Siow D., Megyesi J., Shayman J., Bielawska A., Bielawski J., Beverly L.J. (2017). Inhibiting glucosylceramide synthase exacerbates cisplatin-induced acute kidney injury. J. Lipid Res..

[95] Kong D., Zhuo L., Gao C., Shi S., Wang N., Huang Z., Li W., Hao L. (2013). Erythropoieti protects against cisplatin-induced nephrotoxicity by attenuating endoplasmic reticulum stress-induced apoptosis. J. Nephrol..

[96] Mapuskar K.A., Wen H., Holanda D.G., Rastogi P., Steinbach E., Han R., Coleman M.C., Attanasio M., Riley D.P., Spitz D.R. (2019). Persistent increase in mitochondrial superoxide mediates cisplatin-induced chronic kidney disease. Redox Biol..

[97] Tang J., Shi Y., Liu N., Xu L., Zang X., Li P., Zhang J., Zheng X., Qiu A., Zhuang S. (2018). Blockade of histone deacetylase 6 protects against cisplatin-induced acute kidney injury. Clin. Sci..

[98] Perico L., Morigi M., Rota C., Breno M., Mele C., Noris M., Introna M., Capelli C., Longaretti L., Rottoli D. (2017). Human mesenchymal stromal cells transplanted into mice stimulate renal tubular cells and enhance mitochondrial function. Nat. Commun..

[99] Volarevic V., Djokovic B., Gazdic M.J., Harrell R., Fellabaum C., Djono V., Arsenijevic N. (2019). Molecular mechanisms of cisplatin-induced nephrotoxicity: A balance on the knife edge between renoprotection and tumor toxicity. J. Biomed. Sci..

[100] Hosohata K. (2016). Role of oxidative stress in drug-induced kidney injury. Int. J. Mol. Sci..

[101] Peres L.A., da Cunha A.D. (2013). Acute nephrotoxicity of cisplatin: Molecular mechanisms. J. Bras. Nefrol..

[102] Ibrahim A., Al-Hizab F.A., Abushouk A.I., Abdel-Daim M.M. (2018). Nephroprotective E_ects of Benzyl Isothiocyanate and Resveratrol Against Cisplatin-Induced Oxidative Stress and Inflammation. Front. Pharmacol..

[103] Qi Z., Li Z., Li W., Liu Y., Wang C., Lin H., Liu J., Li P. (2018). Pseudoginsengenin DQ Exhibits Therapeutic E_ects in Cisplatin-Induced Acute Kidney Injury via Sirt1/NF-kappaB and Caspase Signaling Pathway without Compromising Its Antitumor Activity in Mice. Molecules.

[104] Wang Z., Li Y.F., Han X.Y., Sun Y.S., Zhang L.X., Liu W., Liu X.X., Li W., Liu Y.Y. (2018). Kidney Protection E_ect of Ginsenoside Re and Its Underlying Mechanisms on Cisplatin-Induced Kidney Injury. Cell. Physiol. Biochem..

[105] Oh G.S., Kim H.J., Shen A., Lee S.B., Yang S.H., Shim H., Young Cho E., Beom Kwon K., Hwan Kwak T., So H.S. (2016). New Therapeutic Concept of NAD Redox Balance for Cisplatin Nephrotoxicity. BioMed Res. Int..

[106] Srivastava S., Sinha D., Saha P.P., Marthala H., D’Silva P. (2014). Magmas functions as a ROS regulator and provides cytoprotection against oxidative stress-mediated damages. Cell Death Dis..

[107] Tang C., Dong Z. (2016). Mitochondria in Kidney Injury: When the Power Plant Fails. J. Am. Soc. Nephrol..

[108] Hall A.M., Schuh C.D. (2016). Mitochondria as therapeutic targets in acute kidney injury. Curr. Opin. Nephrol. Hypertens..

[109] Oh G.S., Kim H.J., Choi J.H., Shen A., Choe S.K., Karna A., Lee S.H., Jo H.J., Yang S.H., Kwak T.H. (2014). Pharmacological activation of NQO1 increases NAD(+) levels and attenuates cisplatin-mediated acute kidney injury in mice. Kidney Int..

[110] Perse M., Veceric-Haler Z. (2018). Cisplatin-Induced Rodent Model of Kidney Injury: Characteristics and Challenges. BioMed Res. Int..

[111] Zhang B., Ramesh G., Norbury C.C., Reeves W.B. (2007). Cisplatin-induced nephrotoxicity is mediated by tumor necrosis factor-alpha produced by renal parenchymal cells. Kidney Int..

[112] Mukhopadhyay P., Horváth B., Kechrid M., Tanchian G., Rajesh M., Naura A.S., Boulares A.H., Pacher P. (2011). Poly (ADP-ribose) polymerase-1 is a key mediator of cisplatin-induced kidney inflammation and injury. Free Radic. Biol. Med..

[113] Inoue T. (2017). M1 macrophage triggered by Mincle leads to a deterioration of acute kidney injury. Kidney Int..

[114] Summers S.A., Chan J., Gan P.Y., Dewage L., Nozaki Y., Steinmetz O.M., Nikolic-Paterson D.J., Kitching A.R., Holdsworth S.R. (2011). Mast cells mediate acute kidney injury through the production of TNF. J. Am. Soc. Nephrol..

[115] Soni H., Kaminski D., Gangaraju R., Adebiyi A. (2018). Cisplatin-induced oxidative stress stimulates renal Fas ligand shedding. Ren. Fail..

[116] Yang L., Brooks C.R., Xiao S., Sabbisetti V., Yeung M.Y., Hsiao L.L., Ichimura T., Kuchroo V., Bonventre J.V. (2015). KIM-1-mediated phagocytosis reduces acute injury to the kidney. J. Clin. Investig..

[117] Endre Z.H. (2014). Recovery from Acute Kidney Injury: The Role of Biomarkers. Nephron Clin. Pract..

[118] Liu X., Guan Y., Xu S., Li Q., Sun Y., Han R., Jiang C. (2016). Early Predictors of Acute Kidney Injury: A Narrative Review. Kidney Blood Press. Res..

[119] Rizo-Topete L.M., Rosner M.H., Ronco C. (2017). Acute Kidney Injury Risk Assessment and the Nephrology Rapid Response Team, Role of the Nephrologist in Multidisciplinary Management of AKI. Blood Purif..

[120] Nozaki Y., Kinoshita K., Hino S., Yano T., Niki K., Hirooka Y., Kishimoto K., Funauchi M., Matsumura I. (2015). Signaling Rho-kinase mediates inflammation and apoptosis in T cells and renal tubules in cisplatin nephrotoxicity. Am. J. Physiol. Ren. Physiol..

[121] Sinha V., Vence L.M., Salahudeen A.K. (2013). Urinary tubular proteinbased biomarkers in the rodent model of cisplatin nephrotoxicity: A comparative analysis of serum creatinine, renal histology, and urinary KIM-1, NGAL, and NAG in the initiation, maintenance, and recovery phases of acute kidney injury. J. Investig. Med..

[122] Shinke H., Masuda S., Togashi Y., Ikemi Y., Ozawa A., Sato T., Kim Y.H., Mishima M., Ichimura T., Bonventre J.V. (2015). Urinary kidney injury molecule-1 and monocyte chemotactic protein-1 are noninvasive biomarkers of cisplatin-induced nephrotoxicity in lung cancer patients. Cancer Chemother. Pharmacol..

[123] Faig J., Haughton M., Taylor R.C., D’Agostino R.B., Whelen M.J., Rodriguez K.A., Bonomi M., Murea M., Porosnicu M. (2018). Retrospective analysis of cisplatin nephrotoxicity in patients with head and neck Cancer receiving outpatient treatment with concurrent high-dose cisplatin and radiotherapy. Am. J. Clin. Oncol..

[124] Prasaja Y., Sutandyo N., Andrajati R. (2015). Incidence of cisplatin-induced nephrotoxicity and associated factors among cancer patients in Indonesia. Asian Pac. J. Cancer Prev..

[125] Tekce B.K., Uyeturk U., Tekce H., Uyeturk U., Aktas G., Akkaya A. (2015). Does the kidney injury molecule-1 predict cisplatin-induced kidney injury in early stage?. Ann. Clin. Biochem..

[126] Abdelsalam M., Elmorsy E., Abdelwahab H. (2018). Urinary biomarkers for early detection of platinum based drugs induced nephrotoxicity. BMC Nephrol..

[127] Oh S.M., Park G., Lee S.H., Seo C.S., Shin H.K., Oh D.S. (2017). Assessing the recovery from prerenal and renal acute kidney injury after treatment with single herbal medicine via activity of the biomarkers HMGB1, NGAL and KIM-1 in kidney proximal tubular cells treated by cisplatin with different doses and exposure times. BMC Complement. Altern. Med..

[128] McMahon K.R., Rod Rassekh S., Schultz K.R. (2017). Design and Methods of the Pan-Canadian Applying Biomarkers to Minimize Long-Term Effects of Childhood/Adolescent Cancer Treatment (ABLE) Nephrotoxicity Study: A Prospective Observational Cohort Study. Can. J. Kidney Health Dis..

[129] Salem N., Helmi N., Assaf N. (2018). Renoprotective Effect of Platelet-Rich Plasma on Cisplatin-Induced Nephrotoxicity in Rats. Oxid. Med. Cell. Longev..

[130] Jun D.Y., Kim S.Y., Na J.C., Lee H.H., Kim J., Yoon Y.E., Hong S.J., Han W.K. (2018). Tubular organotypic culture model of human kidney. PLoS ONE.

[131] Huang J.X., Kaeslin G., Ranall M.V. (2015). Evaluation of biomarkers for in vitro prediction of druginduced nephrotoxicity: Comparison of HK-2, immortalized human proximal tubule epithelial, and primary cultures of human proximal tubular cells. Pharmacol. Res. Perspect..

[132] Wang X., Grunz-Borgmann E.A., Parrish A.R. (2014). Loss of _(E)-Catenin Potentiates Cisplatin-Induced Nephrotoxicity via Increasing Apoptosis in Renal Tubular Epithelial Cells. Toxicol. Sci..

[133] Gardiner L., Akintola A., Chen G., Catania J.M., Vaidya V., Burghardt R.C., Bonventre J.V., Trzeciakowski J., Parrish A.R. (2012). Structural equation modeling highlights the potential of Kim-1 as a biomarker for chronic kidney disease. Am. J. Nephrol..

[134] Timothy J., Piantaa B., John W., Succar P.L., Chin M., Davidson T., Buckley N.A., Mohamed F., Endre Z.H. (2017). Dexamethasone Modifies Cystatin C-Based Diagnosis of Acute Kidney Injury During Cisplatin-Based Chemotherapy. Kidney Blood Press. Res..

[135] Meng H., Fu G., Shen J., Shen K., Xu Z., Wang Y., Jin B., Pan H. (2017). Ameliorative Effect of Daidzein on Cisplatin-Induced Nephrotoxicity in Mice via Modulation of Inflammation, Oxidative Stress, and Cell Death. Oxid. Med. Cell. Longev..

[136] McDuffie J.E., Chen Y., Ma J.Y., Lee K.M., Lynch D.M., Hamlin L., Nguyen M., Rizzolio M., Soneed M., Snooka S. (2016). Cisplatin nephrotoxicity in male beagle dogs: Next-generation protein kidney safety biomarker tissue expression and related changes in urine. Toxicol. Res..

[137] Nishihara K., Masuda S., Shinke H., Ozawa A., Ichimura T., Yonezawa A., Nakagawa S., Inui K., Bonventre J.V., Matsubara K. (2013). Urinary chemokine (C-C motif) ligand 2 (monocyte chemotactic protein-1) as a tubular injury marker for early detection of cisplatin-induced nephrotoxicity. Biochem. Pharmacol..

[138] DesRochers T.M., Suter L., Roth A., Kaplan D.L. (2013). Bioengineered 3D Human Kidney Tissue, a Platform for the Determination of Nephrotoxicity. PLoS ONE.

[139] Rysz J., Gluba-Brzózka A., Franczyk B., Jabłonowski Z., Ciałkowska-Rysz A. (2017). Novel Biomarkers in the Diagnosis of Chronic Kidney Disease and the Prediction of Its Outcome. Int. J. Mol. Sci..

[140] Vinken P., Starckx S., Barale-Thomas E., Looszova A., Sonee M., Goeminne N., Vermissen L., Buyens K., Lampo A. (2012). Tissue Kim-1and urinary clusterin as early indicators of cisplatin-induced acute kidney injury in rats. Toxicol. Pathol..

[141] Hany H., Araba B., Samir A., Salamaa C., Maghrabi I.A. (2018). Camel Milk Ameliorates 5-Fluorouracil- Induced Renal Injury in Rats: Targeting MAPKs, NF-κB and PI3K/Akt/eNOS Pathways. Cell. Physiol. Biochem..

[142] Abdelrahman A.M., Al Suleimani Y., Shalaby A., Ashique M., Manoj P., Al-Saadi H., Ali B.H. (2019). Effect of levosimendan, a calcium sensitizer, on cisplatin-induced nephrotoxicity in rats. Toxicol. Rep..

[143] Sharp C.N., Siskind L.J. (2017). Developing better mouse models to study cisplatin-induced kidney injury. Am. J. Physiol. Ren. Physiol..

[144] Ali B.H., Abdelrahman A.M., Al-Salam S., Sudhadevi M., AlMahruqi A.S., Al-Husseni I.S., Beegam S., Dhanasekaran S., Nemmar A., Al-Moundhri M. (2011). The effect of sildenafil on cisplatin nephrotoxicity in rats. Basic Clin. Pharmacol. Toxicol..

[145] Al Suleimani Y.M., Abdelrahman A.M., AlMahruqi A.S., Alhseini I.S., Tageldin M.H., Mansour M.E., Ali B.H. (2010). Interaction of nimesulide, a cyclooxygenase- 2 inhibitor, with cisplatin in normotensive and spontaneously hypertensive rats. Food Chem. Toxicol..

[146] Khames A., Khalaf M.M., Gad A.M., Abd El-Raouf O.M., Kandeil M.A. (2019). Nicorandil combats doxorubicin-induced nephrotoxicity via amendment of TLR4/P38 MAPK/NFκ-B signaling pathway. Chem. Biol. Interact..

[147] Khan T.H., Ganaie M.A., Alharthy K.M., Madkhali H., Jan B.L., Sheikh I.A. (2018). Naringenin prevents doxorubicin-induced toxicity in kidney tissues by regulating the oxidative and inflammatory insult in Wistar rats. Arch. Physiol. Biochem..

[148] Rashid S., Ali N., Nafees S., Hasan S.K., Sultana S. (2014). Mitigation of 5-Fluorouracil induced renal toxicity by chrysin via targeting oxidative stress and apoptosis in wistar rats. Food Chem. Toxicol..

[149] Vaidya V.S., Ford G.M., Waikar S.S., Wang Y., Clement M.B., Ramirez V., Glaab W.E., Troth S.P., Bonventre J.V. (2009). A rapid urine test for early detection of kidney injury. Kidney Int..

[150] Sabbisetti V.S., Ito K., Wang C., Mefferd S.C., Bonventre J.V. (2013). Novel Assays for Detection of Urinary KIM-1 in Mouse Models of Kidney Injury. Toxicol. Sci..

[151] Ragab D., Abdallah D.M., El-Abhar H.S. (2014). Cilostazol Renoprotective Effect: Modulation of PPAR-c, NGAL, KIM-1 and IL-18 Underlies Its Novel Effect in a Model of Ischemia-Reperfusion. PLoS ONE.

[152] Xu J., Sun L., Sun W., Tian J., Guo H. (2018). Targeted Silencing of *Kim-1* Inhibits the Growth of Clear Cell Renal Cell Carcinoma Cell Line 786-0 In Vitro and in Vivo. Oncol. Res..

[153] Hayati F., Hossainzadeh M., Shayanpour S., Abedi-Gheshlaghi Z., Beladi Mousavi S.S. (2016). Prevention of cisplatin nephrotoxicity. J. Nephropharmacol..

[154] Crona D.J., Faso A., Nishijima T.F., McGraw K.A., Galsky M.D., Milowsky M.I. (2017). A systematic review of strategies to prevent cisplatin-induced nephrotoxicity. Oncologist.

[155] Rafieian-Kopaei M., Baradaran A., Rafieian M. (2013). Plants antioxidants: From laboratory to clinic. J. Nephropathol..

[156] Hemati S., Arbab Jolfaie N., Rafienia M., Ghavamnasiri M. (2012). The effects of vitamin E and selenium on cisplatininduced nephrotoxicity in cancer patients treated with cisplatin-based chemotherapy: A randomized, placebo-controlled study. J. Res. Med. Sci..

[157] Abdel-Daim M.M., Abushouk A.I., Donia T., Alarifi S., Alkahtani S., Aleya L., Bungau S.G. (2019). The nephroprotective effects of allicin and ascorbic acid against cisplatin-induced toxicity in rats. Environ. Sci. Pollut. Res..

[158] Ridzuan N.R., Rashid N.A., Othman F., Budin S.B., Hussan F., Teoh S.L. (2019). Protective Role of Natural Products in Cisplatin-Induced Nephrotoxicity. Mini Rev. Med. Chem..

[159] Cascella M., Palma G., Barbieri A., Bimonte S., Amruthraj N.J., Muzio M.R., Del Vecchio V., Rea D., Falco M., Luciano A. (2017). Role of *Nigella sativa* and Its Constituent Thymoquinone on Chemotherapy-Induced Nephrotoxicity: Evidences from Experimental Animal Studies. Nutrients.

[160] Hosseinian S., Khajavi Rad A., Hadjzadeh M.A.R., Mohamadian Roshan N., Havakhah S., Shafiee S. (2016). The protective effect of *Nigella sativa* against cisplatin-induced nephrotoxicity in rats. Avicenna J. Phytomed..

[161] Santos N.A., Bezerra C.S., Martins N.M., Curti C., Bianchi M.L., Santos A.C. (2008). Hydroxyl radical scavenger ameliorates cisplatin-induced nephrotoxicity by preventing oxidative stress, redox state unbalance, impairment of energetic metabolism and apoptosis in rat kidney mitochondria. Cancer Chemother. Pharmacol..

[162] Lynch E.D., Gu R., Pierce C., Kil J. (2005). Reduction of acute cisplatin ototoxicity and nephrotoxicity in rats by oral administration of allopurinol and ebselen. Hear. Res..

[163] Hensley M.L., Hagerty K.L., Kewalramani T., Green D.M., Meropol N.J., Wasserman T.H., Cohen G.I., Emami B., Gradishar W.J., Mitchell R.B. (2009). American Society of Clinical Oncology 2008 clinical practice guideline update: Use of chemotherapy and radiation therapy protectants. J. Clin. Oncol..

[164] Mousavi S.S., Zadeh M.H., Shahbazian H., Khanzadeh A., Hayati F., Ghorbani A., Golzari K., Valavi E., Motemednia F., Mousavi M.B. (2014). The protective effect of theophylline in cisplatin nephrotoxicity. Saudi J. Kidney Dis. Transpl..

[165] Katsuda H., Yamashita M., Katsura H., Yu J., Waki Y., Nagata N., Miyamoto K. (2010). Protecting cisplatin-induced nephrotoxicity with cimetidine does not affect antitumoractivity. Biol. Pharm. Bull..

[166] Mercantepe F., Mercantepe T., Topcu A., Ylmaz A., Tumkaya L. (2018). Protective effects of amifostine, curcumin, and melatonin against cisplatin- induced acute kidney injury Naunyn-Schmiedeberg’s. Arch. Pharmacol..

[167] Wei L., Chen W., Zou Y., Huang H., Pan B., Jin S., Huang R., Nie S., Kong G. (2015). AMP-activated protein kinase regulates autophagic protection against cisplatin-induced tissue injury in the kidney. Genet. Mol. Res..

[168] Gaião S.M., Paiva J.A.O.D.C. (2017). Biomarkers of renal recovery after acute kidney injury. Rev. Bras. Ter. Intensiva.

